# Fighting against COVID-19 in Vietnam: The value of rapid antibody testing should not be confused

**DOI:** 10.34172/hpp.2020.28

**Published:** 2020-07-12

**Authors:** Minh Van Hoang

**Affiliations:** Hanoi University of Public Health, Hanoi, Vietnam

## Dear Editor,

Vietnam, a middle-income country with more than 96 million population, has mobilized the entire political system in the country to implement a proactive and comprehensive campaign to fight the Coronavirus disease 2019 (COVID-19) pandemic. Employing the principle “Early detection, strict quarantine, isolation as well as an active treatment”, Vietnam has achieved initial success in containing the spread of the disease.^1^ As of April 26, 2020, the number of confirmed COVID-19 cases was 270, 225 recoveries and no deaths.^2^

Vietnam has recently deployed a massive COVID-19 testing campaign, starting in Hanoi capital, using a rapid antibody testing technique, for all people who have been supposedly exposed to someone who might have the disease. However, public concerns about the accuracy of the rapid testing technique used were extensively raised when all the 3 positive cases (out of 753 people tested) by the rapid testing technique got negative results by the World Health Organization approved polymerase chain reaction (PCR) method.

In fact, the inconsistency in these test results can be explained by the difference in the nature of the rapid antibody testing (detecting the antibody against the disease) and the PCR method (detecting the antigen or the active existence of the virus). In fact, many people who had recovered (no more antigen) but still had antibodies against the virus in their blood. This means that the value of rapid antibody testing should not be confused. This also implies that negatively tested cases by the rapid antibody testing technique should not think they definitely have no disease and should not relax their attention to apply isolation and social distancing measures. Newly infected patients, who have no or have a low level of antibody in the blood, would have negative test results.

In summary, the rapid antibody testing technique for COVID-19 should not be misunderstood as a method for diagnosing cases of active virus infection. This can only help to determine who has ever been infected and who may be immune to re-infection which is useful for guiding interventions such as social distancing measures, etc. Proactive and comprehensive preparedness and response to tackle the COVID-19 pandemic should be further strengthened by communication activities about the value of different testing methods.

## Ethical approval

Not applicable.

## Competing interests

The author declares that he has no competing interests.
